# *Aureobasidium melanogenum* isolation from the cerebrospinal fluid of a patient with human immunodeficiency virus/acquired immunodeficiency syndrome: A novel report

**DOI:** 10.1590/0037-8682-0293-2021

**Published:** 2021-08-20

**Authors:** Lincoln de Oliveira Sant’Anna, Louisy Sanches dos Santos, Max Roberto Batista Araújo

**Affiliations:** 1 Universidade do Estado do Rio de Janeiro, Faculdade de Ciências Médicas, Departamento de Microbiologia, Imunologia e Parasitologia, Rio de Janeiro, RJ, Brasil.; 2 Instituto Hermes Pardini, Núcleo Técnico Operacional, Setor de Microbiologia, Vespasiano, MG, Brasil.

**FIGURE 1:**
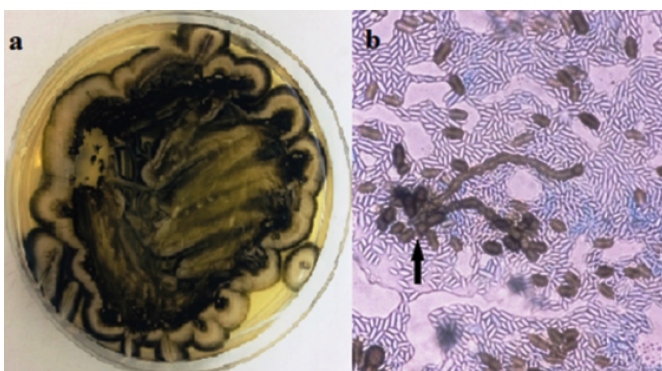
(a): Macromorphology of *A. melanogenum* in Sabouraud Dextrose agar incubated for 7 days at 25 ºC. (b): Image showing dark brown conidia of *A. melanogenum* (black arrow) on a wet mount microscopy slide (40× objective lens).

A 22-year-old Brazilian man with untreated ulcerative colitis and human immunodeficiency virus (HIV) infection was admitted to the intensive care unit (ICU) for holocranial headache, fever, nausea, malaise, and diarrhea. Antiretroviral therapy (ART) was discontinued eight months earlier. Medical history included prior hospitalization due to pneumomediastinum secondary to perforated esophageal moniliasis. Laboratory tests showed leukocytosis, elevated C-reactive protein levels, high HIV viral load (133.627 copies/mL), low CD4+T-cell count (14 cells/mm^3^), and abnormal levels of cerebrospinal fluid (CSF) proteins and glucose. Treatment with ceftriaxone, metronidazole, and mebendazole was initiated. Further CSF analysis was negative for *Cryptococcus* spp., but fungal culture showed the growth of black, rough colonies (Figure 1a) with conidia as observed by optical microscopy (Figure 1b). The fungus was identified as *Aureobasidium melanogenum* using gene sequencing. Treatment with amphotericin B lipid complex was initiated for 14 days, and he was discharged from the ICU after re-initiating ART. 

*A. melanogenum* is ubiquitous in the environment. Although commonly considered a contaminant, this species has been increasingly associated with invasive infections in immunocompromised patients and seems to present a high pathogenic potential^1^ ^-^ ^3^.

The prognosis of *A. melanogenum* infections depends on the extent of infection and host conditions^1^ and on accurate species identification and therapy. To the best of our knowledge, this is the first report of *A. melanogenum* isolation from the CSF of a patient with acquired immunodeficiency syndrome (AIDS). Although such cases in HIV/AIDS patients are rare^1^ ^,^ ^2^, physicians should be aware of the possibility of *A. melanogenum* infections in these individuals.
